# Interpretable deep neural network for cancer survival analysis by integrating genomic and clinical data

**DOI:** 10.1186/s12920-019-0624-2

**Published:** 2019-12-23

**Authors:** Jie Hao, Youngsoon Kim, Tejaswini Mallavarapu, Jung Hun Oh, Mingon Kang

**Affiliations:** 1 Department of Biostatistics, Epidemiology and Informatics, University of Pennsylvania, Philadelphia, PA USA; 2 Department of Computer Science, Kennesaw State University, Marietta, GA USA; 3 Analytics and Data Science Institute, Kennesaw State University, Kennesaw, GA USA; 4 Department of Medical Physics, Memorial Sloan Kettering Cancer Center, New York, NY USA; 5 Department of Computer Science, University of Nevada, Las Vegas, Las Vegas, NV USA

**Keywords:** Cox-PASNet, Deep neural network, Survival analysis, Glioblastoma multiforme, Ovarian cancer

## Abstract

**Background:**

Understanding the complex biological mechanisms of cancer patient survival using genomic and clinical data is vital, not only to develop new treatments for patients, but also to improve survival prediction. However, highly nonlinear and high-dimension, low-sample size (HDLSS) data cause computational challenges to applying conventional survival analysis.

**Results:**

We propose a novel biologically interpretable pathway-based sparse deep neural network, named Cox-PASNet, which integrates high-dimensional gene expression data and clinical data on a simple neural network architecture for survival analysis. Cox-PASNet is biologically interpretable where nodes in the neural network correspond to biological genes and pathways, while capturing the nonlinear and hierarchical effects of biological pathways associated with cancer patient survival. We also propose a heuristic optimization solution to train Cox-PASNet with HDLSS data. Cox-PASNet was intensively evaluated by comparing the predictive performance of current state-of-the-art methods on glioblastoma multiforme (GBM) and ovarian serous cystadenocarcinoma (OV) cancer. In the experiments, Cox-PASNet showed out-performance, compared to the benchmarking methods. Moreover, the neural network architecture of Cox-PASNet was biologically interpreted, and several significant prognostic factors of genes and biological pathways were identified.

**Conclusions:**

Cox-PASNet models biological mechanisms in the neural network by incorporating biological pathway databases and sparse coding. The neural network of Cox-PASNet can identify nonlinear and hierarchical associations of genomic and clinical data to cancer patient survival. The open-source code of Cox-PASNet in PyTorch implemented for training, evaluation, and model interpretation is available at: https://github.com/DataX-JieHao/Cox-PASNet.

## Background

Understanding the complex biological mechanisms of cancer patient survival using genomic and clinical data is vital, not only to develop new treatments for patients, but also to improve survival prediction [1]. As advanced molecular high-throughput sequencing platforms efficiently produce high-dimensional genomic data (e.g., gene expression data and RNA-seq), molecular profiles of human diseases (e.g., cancer) can be obtained [2]. High-dimensional biological data have been increasingly utilized for elucidating their underlying biological mechanisms, as well as supporting clinical decision-making.

Survival analysis is a group of methods used for estimating survival distribution from data, in which the outcome is the survival time until the observation has an event of interest. In survival analysis, it is important to handle right-censoring data, which are another type of missing values. The most prevalent approach for analyzing time-to-event data in clinical trials is the Cox Proportional Hazards regression model (Cox-PH) [3, 4]. It is a semi-parametric model, which has few assumptions, but is effective to interpret the effects between risk factors. For instance, both conventional and stratified Cox models were applied for analyzing more than 15,000 patients who have breast cancer, so as to assess the association between cancer treatments and survival time, as well as cancer stage [5]. Furthermore, a Cox-PH model was performed with about 400 breast cancer patients, and it was discovered that chronic diseases affected cancer patient survival [6].

However, the main obstacles in the conventional Cox-PH model are (1) analyzing high-dimension, low-sample size (HDLSS) data; and (2) handling the highly nonlinear relationship between covariates. In bioinformatics, analyzing HDLSS data is essential and challenging, since most biological data have limited samples (*n*) but an extremely large number of features (*p*), i.e., *p*>>*n*. The high-dimensional data often result in, either training infeasible or overfitting of the training dataset [7]. As a consequence, low-dimensional, large-enough sample size data, such as clinical information, are used to apply the conventional Cox-PH model directly for predicting patient survival. Nevertheless, a dramatic rise in research for analyzing high-dimension genomic data has been observed, so as to disclose the effects of the molecular biological mechanism on patient survival. Feature selection methods, such as penalization algorithms, have generally been considered to address the HDLSS issue in the Cox-PH model. Penalty-based Cox-PH models, with LASSO (*L*_1_) or elastic-net regularization, were frequently used for high-dimensional genomic data [8–11]. Additionally, an advanced feature selection approach was proposed to guarantee the selection algorithm included almost all of the significant covariates [12].

The effects of genomic data on patient survival are generally highly nonlinear for complex human diseases [13], but the conventional Cox-PH model assumes the linear contributions of covariates. The kernel trick can explicitly transform nonlinear covariate effects to become linear for linear regression algorithms. A kernel-based Cox-PH model was proposed to handle the nonlinear effects of gene expression profiles on censored survival phenotypes, such as overall survival time and relapse time [14]. Moreover, two survival support vector machine (SVM) models, for both classification and regression problems, were proposed to improve survival prediction with high-dimensional genomic data [15]. It is still challenging to seek the optimal kernel function, with the optimal pair of hyper-parameters, since kernel-based models need to specify the kernel function beforehand.

Deep learning techniques have recently drawn attention in bioinformatics because of their automatic capturing of nonlinear relationships, from their input and a flexible model design. Several deep learning models, which incorporate a standard Cox-PH model as an output layer, have been proposed for predicting patient survival. DeepSurv incorporates a standard Cox-PH regression, along with a deep feed-forward neural network in order to improve survival prediction, and eventually build a recommendation system for personalized treatment [16]. DeepSurv has achieved competitive performance, compared to standard Cox-PH alone and random survival forests (RSFs). However, the limitation of DeepSurv is that only very low-dimension clinical data were examined, where the number of variables was less than 20. Cox-nnet, an artificial neural network for a regularized Cox-PH regression problem, was proposed in order to high-throughput RNA sequencing data [17]. Overall, Cox-nnet outperformed a regularized Cox-PH regression (alone), RSF, and CoxBoost. In Cox-nnet, the top-ranked hidden nodes, which are the latent representations from gene expression data, are associated to patient survival, and each hidden node may implicitly represent a biological process. In a similar fashion, SurvivalNet adopted a Bayesian Optimization technique, so as to automatically optimize the structure of a deep neural network [18]. SurvivalNet produced slightly better performance than Cox elastic net (Cox-EN) and RSF. Intriguingly, a well-trained SurvivalNet can generate the risk score for each node by a risk backpropagation analysis.

However, applying deep learning approaches to high-dimensional genomic data for survival analysis is still challenging due to: (1) an overfitting problem when training a deep learning model with HDLSS data; and (2) the lack of explicit model interpretation. Deep neural network models involve a large number of parameters. Thus, deep learning typically requires a large number of samples. Particularly, when training a deep learning model with HDLSS data, gradients tend to have high variance in backpropagation, which consequently causes model overfitting. Both Cox-nnet and SurvivalNet introduced only significant genomic data by feature selection approaches, to avoid the overfitting problem, so the methods may fail to handle high-dimensional data. In order to overcome the HDLSS problem in deep learning, dimension reduction techniques were employed to reduce the dimension of the input data, and the lower dimensional data were introduced to a neural network [19]. Deep Feature Selection was developed to identify discriminative features in a deep learning model [20]. Deep Neural Pursuit trained a small-sized sub-network and computed gradients with low variance for feature selection [21].

Although there are variant architectures in deep learning, most conventional deep neural networks consist of multiple fully-connected layers for analyzing structure data, which make them difficult to interpret. In survival analysis, model interpretation (e.g., identifying prognosis factors) is often more important than simply predicting patient survival with high accuracy. However, hidden nodes, computed by fully-connected layers, are not able to represent explicit biological components. Moreover, biological processes may involve only a small number of biological components, rather than all input features. Thus, the capability of explicit model interpretation in deep neural networks is highly desired in survival analysis.

Additionally, the interpretation of hierarchical interactions of biological pathways has barely been addressed. Intuitively, the biological interpretation at a pathway level enables obtaining rich biological findings. This is because a pathway-based analysis usually shows remarkable power in reproducibility with genomic studies. For example, highly reproducible biomarkers have been identified in diagnosing breast cancer by high-level representation of pathway-based metabolic features [22].

Biological systems are often complex, and may include hierarchical interactions between molecular pathways. Different survival rates between patients may be caused by those hierarchical relationships between pathways. In particular, for antiviral signaling, the hierarchical representation between receptor pathways and gene ontology was explored [23]. Consequently, a deep learning model can be biologically interpretable by incorporating the impacts of inhibition and propagation between pathways.

The integration of multiple types of data (e.g., multi-omics data or clinical data) in a deep learning model is also challenging. A number of studies have reported that leveraging multi-omics and clinical data improves predictive performance in survival analysis [18, 24, 25]. A naive approach to integrate multi-omics data is to combine all types of data into a single matrix and perform a survival analysis [18, 26]. The approach assumes that the heterogeneous data can be represented by an augmented matrix form. However, the augmented matrix causes problems: (1) it generates a much higher dimension of HDLSS data; (2) it makes the sample size smaller due to missing values; and (3) it ignores data types having smaller numbers of covariates. Note that multi-omics data on The Cancer Genome Atlas (TCGA) present substantial missing values; e.g., 160 samples of mRNA-Seq are available, while 595 clinical samples are in the glioblastoma multiforme (GBM) dataset in TCGA.

In this paper, we develop a novel pathway-based sparse deep neural network, named Cox-PASNet, for survival analysis by integrating high-dimensional genomic data and clinical data. Our main contributions of Cox-PASNet for survival analysis are:

- to identify nonlinear and hierarchical relationships at biological gene and pathway levels;
- to provide a solution for neural network model interpretation, in which each node corresponds to a biological components or process;
- to integrate multiple types of data in a deep learning model; and
- to propose efficient optimization for training a neural network model with HDLSS data to avoid overfitting.

This paper is an expanded version of a paper entitled *Cox-PASNet: Pathway-based Sparse Deep Neural Network for Survival Analysis*, presented at the IEEE International Conference on Bioinformatics & Biomedicine (IEEE BIBM 2018), Madrid, Spain, Dec. 3-6 2018 [27].

## Results

### Datasets

In this study, we considered glioblastoma multiforme (GBM) and ovarian serous cystadenocarcinoma (OV) cancers to assess the performance of Cox-PASNet, the proposed model. GBM is the most aggressive malignant tumor that grows rapidly within brain, and the prognosis performance remains poor [28]; OV cancer is a common type of cancer among women in the world, and it is usually diagnosed at a late stage [29]. We collected gene expression and clinical data for TCGA GBM and OV cancers from cBioPortal (www.cbioportal.org/datasets). The patients who had neither survival time nor event status were excluded.

We obtained biological pathways, seen as the prior knowledge, from the Molecular Signatures Database (MSigDB) [30], where we considered both KEGG and Reactome databases for the pathway-based analysis. We excluded small pathways (i.e., less than fifteen genes) and large pathways (i.e., over 300 genes), since small pathways are often redundant with other larger pathways, and large pathways are related to general biological pathways, rather than specific to a certain disease [31]. Moreover, we investigated the genes that were included in at least one of these pathways.

Additionally, we integrated the clinical information from both the GBM and OV cancer patients. Only age was incorporated in the clinical layer of Cox-PASNet, since age was a significantly strong prognostic factor in GBM [24], and most other corresponding clinical information had a large number of missing data. For instance, the Karnofsky Performance Score (KPS) has been known as another significant factor, in addition to age. However, there is a strong correlation between KPS and age, and many patients lack the KPS information. Finally, we have 5,404 genes, 659 pathways, and clinical age data from 523 GBM patients and 532 OV cancer patients.

### Experimental design

The predictive performance of Cox-PASNet was evaluated by comparing to current state-of-the-art methods, such as Cox-EN [10], Cox-nnet [17], and SurvivalNet [18]. For the measurement of predictive performance with censored data, we considered C-index, which is a rank-correlation method that counts concordant pairs between the predicted score and observed survival time. The C-index is from zero and one, where one means an ideal prediction, and 0.5 indicates a random prediction.

We repeated the holdout evaluation 20 times for the reproducibility of model performance, due to a small number of samples, with the two targets of survival months and censor status (i.e., living and deceased), and computational costs. On each experiment, the dataset was randomly selected: 20% for the test data, and the remaining 80% data were split into training (80%) and validation (20%), while ensuring the same censoring percentage on each training, validation, and test data. For the training data, we normalized the gene expressions and age to zero mean and unit standard deviation. Then we used the corresponded mean and standard deviation values, calculated from the training data, to normalize the validation and test data, so that any information from the test data was not used for training. We trained every model with the training data, and the validation data were applied to find the optimal pair of hyper-parameters. Once the model was well-trained, the test data were used to evaluate the predictive performance.

### Model tuning

Cox-PASNet was developed based on a modern deep learning model. For the activation function, we used the Tanh function, which produced the highest C-index score compared to other activation functions such as ReLU and LeakyReLU. Additionally, Tanh is beneficial because it provides a probabilistic interpretation to indicate a node’s activation. Both dropout and *L*^2^ regularization were considered. Dropout rates were settled on 0.7 and 0.5 in the pathway layer and the first hidden layer, respectively, with an empirical search. For the neural network optimizer, Adaptive Moment Estimation (Adam) was performed [32], where a grid search was applied in order to approximate the optimal learning rate (*η*) and *L*^2^ penalty term (*λ*). On each experiment, the optimal hyper-parameters of *η* and *λ* were chosen to minimize the cost function with the validation data, and then the model was trained with the optimal hyper-parameters. The implementation of Cox-PASNet in the PyTorch framework is freely available at https://github.com/DataX-JieHao/Cox-PASNet.

In order to a nearly fair comparison, we used the *Glmnet Vignette* Python package [10] for the Cox-EN model. The optimal hyper-parameters of *α* and *λ* were found by a grid search, as Cox-PASNet did. The candidates of *α* are in the range [0,1] with a 0.01 stride, and the length of *λ* is 200. Then we trained the Cox-EN model with the optimal hyper-parameters in the training data, and evaluated the model performance with the associated test data. Cox-nnet was trained by following the implementation codes provided by the authors’ GitHub. We used the default tuning setting and applied a grid search for *L*^2^. As for SurvivalNet, we optimized the hyper-parameters by the Bayesian Optimization technique, *BayesOpt*, which was highlighted to automatically optimize the SurvivalNet [33]. We added two additional hyper-parameters, *L*^1^ and *L*^2^ penalty terms, into the *BayesOpt* algorithm, besides their default search. SurvivalNet was conducted based on open source codes provided by the authors’ GitHub.

For integrating two different types of data, both gene expression and clinical age data were augmented into a big input matrix, which was introduced to benchmark models of Cox-EN, Cox-nnet, and SurvivalNet. Meanwhile, we introduced gene expression and clinical age data into the gene and clinical layer, separately.

### Experimental results

**Fig. 1:**
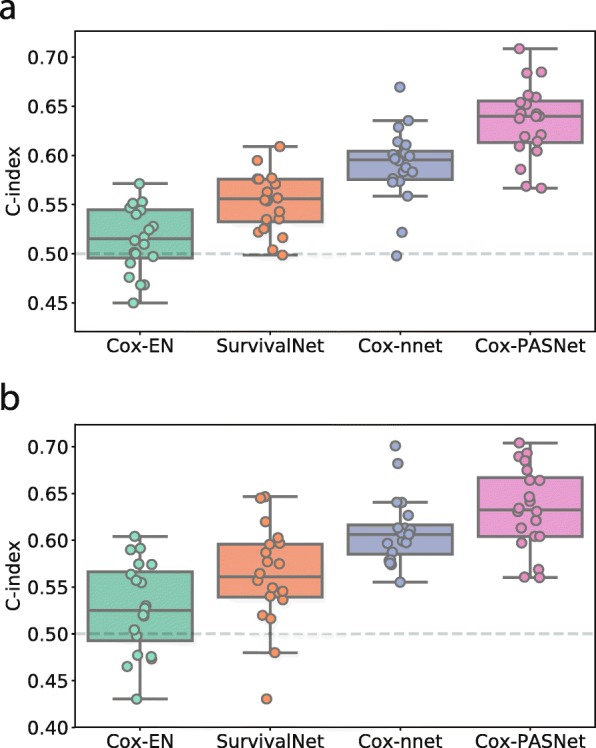
Experimental results with **a** GBM and **b** OV cancer in C-index. Boxplots of C-index of **a** TCGA GBM dataset and **b** TCGA OV cancer dataset using Cox-EN, SurvivalNet, Cox-nnet, and Cox-PASNet. On each experiment, the dataset was randomly selected: 20% for the test data, and the remaining 80% data were split into training (80%) and validation (20%), while ensuring the same censoring percentage on each training, validation, and test data. The experiments were repeated over 20 times

The experimental results with GBM and OV cancer data are shown in Fig. 1 and Tables 1 and 2. With GBM data, our proposed Cox-PASNet obtained the best C-index of 0.6347 ±0.0372, while Cox-nnet was ranked as the second, with a C-index of 0.5903 ±0.0372 (see Fig. 1a and Table 1). Cox-nnet is an artificial neural network that has one hidden layer only. SurvivalNet is a multilayer perceptron, which is an advanced model compared to Cox-nnet, and the optimal architecture of SurvivalNet is ascertained by the *BayesOpt*. Meanwhile, Cox-nnet illustrated that a simpler neural network usually produces a better performance compared to deeper networks [17]. Hence, SurvivalNet produced an average C-index of 0.5521 ±0.0295, which was lower than Cox-nnet’s. Additionally, Cox-EN turned out a C-index of 0.5151 ±0.0336, which was nearly same as a random guess. The poor performance of Cox-EN may be caused by the highly nonlinearity of biological data, which have 5,404 gene expressions but only 523 patients. A Wilcoxon test was run in order to confirm if the outperformance of Cox-PASNet was statistically significant compared to the other three benchmarks. In Table 3, it clearly showed that Cox-PASNet was significantly better than Cox-EN, Cox-nnet, and SurvivalNet, respectively.

**Table 1:** Comparison of C-index with GBM in over 20 experiments

Model	C-index
Cox-EN	0.5151 ±0.0336
Cox-nnet	0.5903 ±0.0372
SurvivalNet	0.5521 ±0.0295
Cox-PASNet	0.6347 ±0.0372

**Table 2:** Comparison of C-index with OV cancer in over 20 experiments

Model	C-index
Cox-EN	0.5276 ±0.0482
Cox-nnet	0.6095 ±0.0356
SurvivalNet	0.5614 ±0.0524
Cox-PASNet	**0.6343 ±0.0439**

Bolded indicates the highest performance

**Table 3:** Statistical assessment with GBM

	Wilcoxon rank-sum test
Cox-PASNet vs. Cox-EN	8.85e-05 ^∗^
Cox-PASNet vs. Cox-nnet	4.49e-4 ^∗^
Cox-PASNet vs. SurvivalNet	1.40e-4 ^∗^

^*^shows the statistical significance with significance level = 0.05

Moreover, we evaluated Cox-PASNet with OV cancer data. Cox-PASNet obtained the best C-index of 0.6343 ±0.0439, as well; Cox-nnet retained the second rank with a C-index of 0.6095 ±0.0356; and Cox-EN was the last place with a C-index of 0.5276 ±0.0482 (Fig. 1b and Table 2). The statistical testing of Wilcoxon test showed that Cox-PASNet also statistically outperformed others in OV cancer in Table 4.

**Table 4:** Statistical assessment with OV cancer

	Wilcoxon rank-sum test
Cox-PASNet vs. Cox-EN	1.03e-4 ^∗^
Cox-PASNet vs. Cox-nnet	0.04 ^∗^
Cox-PASNet vs. SurvivalNet	2.93e-4 ^∗^

^*^shows the statistical significance with significance level = 0.05

It is noted that Cox-PASNet uses the same loss function, which is a negative log partial likelihood, as Cox-EN, Cox-nnet and SurvivalNet. Nevertheless, we leverage a deep neural network architecture with a prior biological knowledge of pathways in Cox-PASNet. The biologically motivated neural network has a better predictive performance, and reduces the noise signals from the complex biological data. Additionally, Cox-PASNet has been trained with small sub-networks, so as to prevent overfitting. Hence, Cox-PASNet makes two contributions of the biological motivated architecture and the new strategy in training, to eventually improve the predictive performance.

## Discussion

### Model interpretation in GBM

**Fig. 2:**
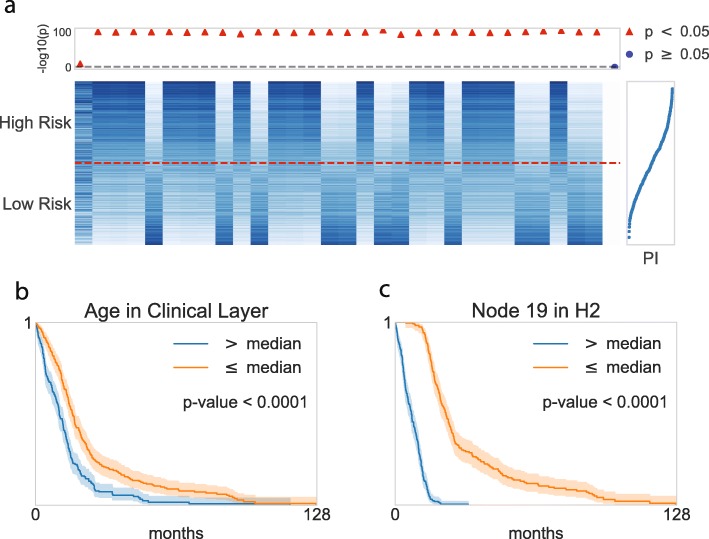
Graphical visualization of the node values in the second hidden layer (H2) and clinical layer. **a** Heatmap of the 31 nodes (i.e., thirty H2 nodes and one clinical node). The horizontal dashed line in red distinguishes two risk groups, where the upper/lower partition belongs to high risk/low risk patients. The top dot plot indicates the nodes’ significance. A logrank test was conducted for each node within two risk groups in the scale of -log10(*p*-values), where red indicates statistical significance, and blue shows insignificance. The plot in the right panel displays the prognostic index (PI) with each corresponding sample. **b**–**c** Kaplan-Meier plots of the top two nodes

**Fig. 3:**
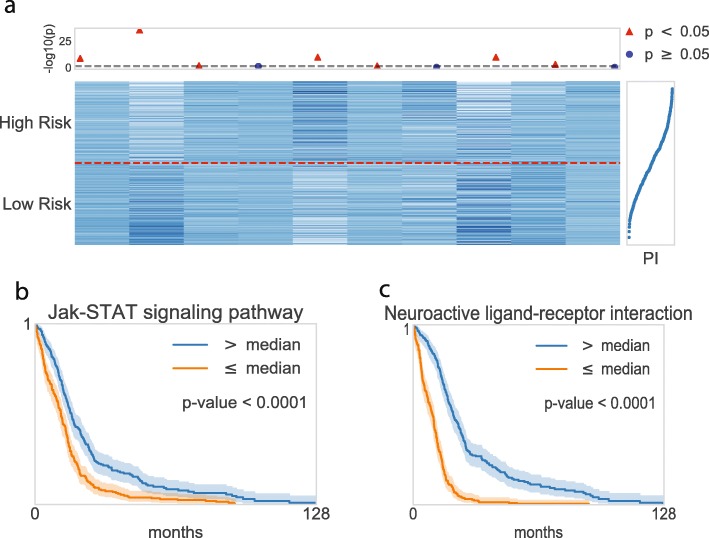
Graphical visualization of the node values in the pathway layer. **a** Heatmap of the top ten pathway nodes. The horizontal dashed line in red distinguishes two risk groups, where the upper/lower partition belongs to high risk/low risk patients. The top dot plot indicates the nodes’ significance. A logrank test was conducted for each node within two risk groups in the scale of -log10(*p*-values), where red indicates statistical significance, and blue shows insignificance. The plot in the right panel displays the prognostic index (PI) with each corresponding sample. **b**–**c** Kaplan-Meier plots for the top two pathway nodes

For the biological model interpretation of Cox-PASNet, we re-trained the model with the optimal pair of hyper-parameters from 20 experiments using all available GBM samples. The samples were categorized into two groups, of high-risk and low-risk, by the median Prognostic Index (PI), which is the output value of Cox-PASNet. The node values of the two groups in the integrative layer (i.e., the second hidden layer (H2) and the clinical layer) and the pathway layer are illustrated in Figs. 2 and 3, respectively. In Fig. 2a, the node values of 31 covariates (30 from the genomic data, and age from the clinical data) were sorted by the average absolute partial derivatives, with respect to the integrative layer. Age (the first column in Fig. 2a) is shown as the most important covariate in Cox-PASNet with GBM data, in terms of the partial derivatives.

The top-ranked covariates show distinct distributions between high-risk and low-risk groups. For instance, the first three covariates in H2 (the 2nd, 3rd, and 4th columns in Fig. 2a) were activated in the high-risk group, but inactivated in the low-risk group. Moreover, we performed a logrank test by grouping the node values of the covariate into two groups individually, again by their medians. The -log10(*p*-values) computed by the logrank test are depicted in the above panel, aligning with the covariates in Fig. 2a. The red triangle markers show significant covariates (-log10(*p*-value) >1.3), whereas the blue markers show insignificant ones. The logrank tests revealed that the top-ranked covariates by the absolute weight are associated to survival prediction. Figure 2b-c present Kaplan-Meier curves for the top two covariates, where survivals between the two groups are significantly different. Thus, the top-ranked covariates can be considered as prognostic factors.

In the same manner, the nodes in the pathway layer are partially illustrated in Fig. 3. The heatmap in Fig. 3a depicts the top 10 pathway node values of the high-risk and low-risk groups, where the pathway nodes are sorted by the average absolute partial derivatives, with respect to the pathway layer. We also performed logrank tests on each pathway node, and 304 out of 659 pathways were statistically significant on the survival analysis. The two top-ranked pathways were further investigated by a Kaplan-Meier analysis, shown in Fig. 3b-c. The Kaplan-Meier curves of the two top-ranked pathways imply the capability of the pathway nodes as prognostic factors.

**Fig. 4:**
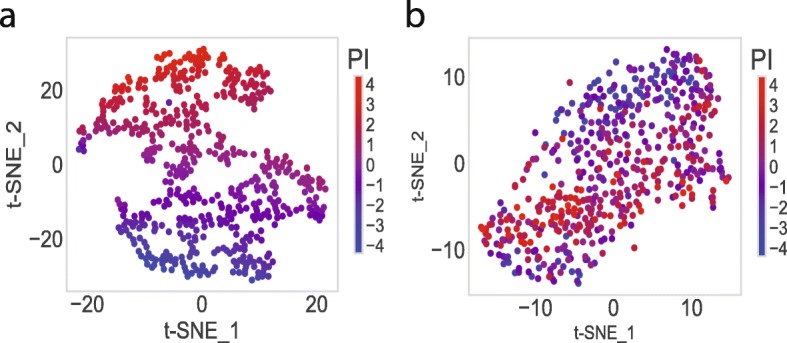
Visualization of the top-ranked nodes by Cox-PASNet. **a** t-SNE plots of the statistically significant nodes in the integrative layer (i.e. the second hidden layer (H2) and clinical layer) and **b** t-SNE plots of the top ten pathway nodes

The statistically significant nodes in the integrative layer, and the top ten ranked pathway nodes, are visualized by t-SNE [34] in Fig. 4, respectively. The nonlinearity of the nodes associated with PI is illustrated. The integrative layer represents the hierarchical and nonlinear combinations of pathways. Thus, the more distinct associations with survivals are shown in the integrative layer than the pathway layer.

The ten top-ranked pathways, with related literature, are listed in Table 5. The *p*-values in the table were computed by a logrank test with the pathway node values of the two groups of high and low risks. Among them, five pathways were reported as significant in the biological literature of GBM. The Jak-STAT signaling pathway, which is usually called an oncopathway, is activated for the tumor growth of many human cancers [35]. Inhibition of the Jak-STAT signaling pathway can reduce malignant tumors, using animal models of glioma. A neuroactive ligand-receptor interaction was explored as one of the most significant pathways in GBM [38]. PI3K cascade is also a well-known pathway, which is highly involved in proliferation, invasion, and migration in GBM [39].

**Table 5:** Ten top-ranked pathways in GBM by Cox-PASNet

Pathway name	Size	*P*-value	Ref.
Jak-STAT signaling pathway	155	<0.0001	[35–37]
Neuroactive ligand-receptor interaction	272	<0.0001	[38]
MAP kinase activation in TLR cascade	50	0.0176	–
NF *κ*B and MAP kinases activation mediated by TLR4 signaling repertoire	72	0.0729	–
G alpha (i) signalling events	195	<0.0001	–
PI3K cascade	71	0.0304	[39, 40]
Tyrosine metabolism	42	0.5671	–
Neuronal system	279	<0.0001	[41]
Axon guidance	129	0.0012	[37]
Xenobiotics	16	0.6347	–

The ten top-ranked genes, by partial derivatives with respect to each gene, are listed with their *p*-values, and related literature, in Table 6. PRL has been known to be associated with the occurrence of neoplasms and central nervous system neoplasms, and so an assessment with PRL expression in primary central nervous system tumors was investigated [42]. MAPK9 was identified as a novel potential therapeutic marker, along with RRM2 and XIAP, which are associated with the biological pathways involved in the carcinogenesis of GBM [43]. IL22 was reported to promote the malignant transformation of bone marrow-derived mesenchymal stem cells, which exhibit potent tumoritropic migratory properties in tumor treatment [44]. FGF5 contributes to the malignant progression of human astrocytic brain tumors as an oncogenic factor in GBM [45]. The activation of JUN, along with HDAC3 and CEBPB, may form resistance to the chemotherapy and radiation therapy of hypoxic GBM; and the downregulation of the genes appeared to inhibit temozolomide on hypoxic GBM cells [46]. A low expression of DRD5 was presented as being associated with relatively superior clinical outcomes in glioblastoma patients with ONC201 [47]. HTR7, involved in neuroactive ligand-receptor interaction and the calcium signaling pathway, was reported to contribute to the development and progression of diffuse intrinsic pontine glioma [48].

**Table 6:** Ten top-ranked genes in GBM by Cox-PASNet

Gene name	*P*-value	Ref.
PRL	0.1698	[42]
FGF22	0.4503	–
MAPK9	0.9580	[43]
IL22	0.0140	[44]
IFNA5	0.5401	–
FGF5	<0.0001	[45]
AGTR1	0.1375	–
JUN	0.1798	[46]
DRD5	0.1288	[47]
HTR7	0.7751	[48]

It is worth noting that only IL22 and FGF5 are statistically significant (i.e., *p*-value <0.05) by logrank test on each gene, which means that only these two genes can be identified as significant prognostic factors by conventional Cox-PH models. However, other genes such as PRL, MAPK9, JUN, DRD5, and HTR7 have been biologically identified as significant prognostic factors, even though significantly different distributions are not found in gene expression (i.e., *p*-value ≥0.05). The average absolute partial derivatives, with respect to each gene, measure the contribution to patients’ survival through the pathway and hidden layers in Cox-PASNet, when gene expression varies on the gene. Therefore, the gene biomarker identification by Cox-PASNet allows one to capture significant genes nonlinearly associated to patients’ survival.

**Fig. 5:**
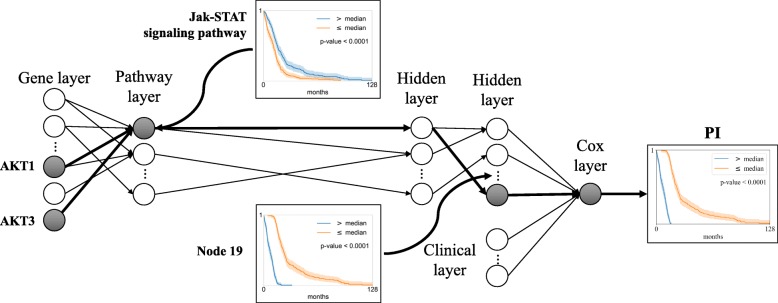
Hierarchical and associational feature representation in Cox-PASNet. For instance, Jak-STAT signaling pathway shows active status, which is associated to PI. The significance of the genes (i.e. AKT1 and AKT3) involved in the Jak-STAT signaling pathway can be ranked by the average absolute partial derivatives with respect to the gene layer. A set of the active pathways are represented in an active Node 19 in the following hidden layers, which improves the survival prediction

Cox-PASNet’s overall model interpretation and hierarchical representations in gene and biological pathway levels are illustrated in Fig. 5. A pathway node represents a latent quantity of the associated gene, and a hidden node expresses the high-level representation of a set of pathways. The following hidden layers describe the hierarchical representation of the previous hidden nodes with sparse connections, which help to identify important pathways and their interactions to contribute to the system. Then, the last hidden nodes are introduced to a Cox-PH model with clinical data.

A pathway node value shows the active or inactive status of the corresponding pathway, which may be associated to different survivals (e.g., Jak-STAT signaling pathway). The significance of the genes involved in the active pathway can be ranked by the absolute weight values between the gene layer and the pathway layer (e.g., AKT1). A set of the active pathways is represented in an active node in the following hidden layer, which improves the survival prediction. For instance, the Kaplan-Meier plots of Node 19 and PI show a more similar estimation of survival than the Jak-STAT signaling pathway, in Fig. 5.

### Limitations

Cox-PASNet captures pathway-based biological mechanisms associated with cancer patients’ survival by embedding pathway databases into the neural network model. Most studies have post-processed pathway-based analysis based on the significant genes identified by their models, whereas in Cox-PASNet, those genes without pathway annotations were not considered in the analysis.

In this study, we considered only GBM and OV cancers in TCGA to evaluate Cox-PASNet. It would be desirable, as future work, to cross validate with genomic data sets other than TCGA for further assessment.

## Conclusion

Deep learning-based survival analysis has been highlighted due to its capability to identify nonlinear prognostic factors and higher predictive performance. However, training deep learning models with high-dimensional data without overfitting and lack of model interpretability in biology were yet-to-be problems. To tackle the challenges, we developed a pathway-based sparse deep neural network, named Cox-PASNet, for survival analysis. Cox-PASNet is a deep learning based model cooupled with a Cox proportional-hazards model that can capture nonlinear and hierarchical mechanisms of biological pathways and identify significant prognostic factors associated to patients’ survival. A new model optimization technique with HDLSS data was introduced to obtain the optimal sparse model without overfitting problem in the paper. We assessed Cox-PASNet with GBM and ovarian cancer data in TCGA. The experimental results showed that Cox-PASNet outperformed the current cutting-edge survival methods, such as Cox-nnet, SurvivalNet, and Cox-EN, and its predictive performance was statistically assessed.

A negative log-partial likelihood with a single node in the output layer is considered in Cox-PASNet, as most deep learning based methods have also done. However, Cox-PASNet constructs the neural network based on biological pathways with sparse coding. The genomic and clinical data are introduced to the model separately for model interpretation.

Cox-PASNet integrates clinical data, as well as genomic data. When combining clinical and genomic data as a large matrix for analysis, the effects of high-dimensional genomic data may dominate the clinical data in the integration, due to the unbalanced size between the genomic and clinical covariates. Cox-PASNet considers separate layers for clinical data and genomic data, so that each data set can be interpreted individually. Furthermore, the incorporation of multi-omics data, such as DNA mutation, copy number variation, DNA methylation, and mRNA expression, is essential to describe complex human diseases involving a sequence of complex interactions in multiple biological processes. A solution for the integration of complex heterogeneous data would also be desirable as future work.

## Methods

### The architecture of Cox-PASNet

**Fig. 6:**
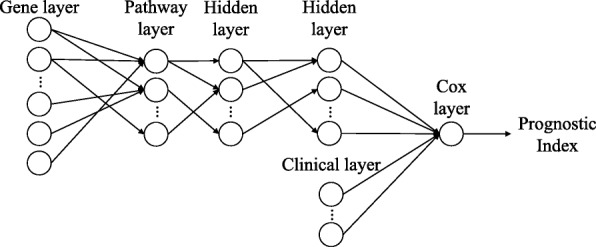
The architecture of Cox-PASNet. The structure of Cox-PASNet is constructed by a gene layer (an input layer), a pathway layer, multiple hidden layers, a clinical layer (additional input layer), and a Cox layer (an output layer)

Cox-PASNet consists of: (1) a gene layer, (2) a pathway layer, (3) multiple hidden layers, (4) a clinical layer, and (5) a Cox layer (see Fig. 6). Cox-PASNet requires two types of ordered data, gene expression data and clinical data from the same patients, where gene expression data are introduced to the gene layer and clinical data are introduced to the clinical layer. The pipeline layers of the two data types are merged in the last hidden layer and produces a Prognostic Index (PI), which is an input to Cox proportional hazards regression. In this study, we included only age as clinical data. Thus, the clinical layer is embedded in the last hidden layer directly, without any additional hidden layers. Higher-dimensional clinical data are desired to be integrated with hidden layers in the clinical pipeline.

#### Gene layer

The gene layer is an input layer of Cox-PASNet, introducing zero-mean gene expression data (**X**) with *n* patient samples of *p* gene expressions, i.e., **X**={**x**_1_,...,**x**_*p*_} and $\mathbf {x}_{i} \sim \mathcal {N}(0, 1)$. For pathway-based analysis, only the genes that belong to at least one pathway are considered in the gene layer.

#### Pathway layer

The pathway layer represents biological pathways, where each node explicitly indicates a specific biological pathway. The pathway layer incorporates prior biological knowledge, so that the neural network of Cox-PASNet can be biologically interpretable. Pathway databases (e.g., KEGG and Reactome) contain a set of genes that are involved in a pathway, and each pathway characterizes a biological process. The knowledge of the given association between genes and pathways, forms sparse connections between the gene layer and the pathway layer in Cox-PASNet, rather than fully-connecting the layers. The node values in the pathway layer measure the corresponding pathways as high-level representations for the survival model.

To implement the sparse connections between the gene and pathway layers, we consider a binary bi-adjacency matrix. Given pathway databases containing pairs of *p* genes and *q* pathways, the binary bi-adjacency matrix ($\mathbf {A} \in \mathbb {B}^{q \times p}$) is constructed, where an element *a*_*ij*_ is one if gene *j* belongs to pathway *i*; otherwise it is zero, i.e., **A**={*a*_*ij*_|1≤*i*≤*q*,1≤*j*≤*p*} and *a*_*ij*_={0,1}.

#### Hidden layers

The hidden layers depict the nonlinear and hierarchical effects of pathways. Node values in the pathway layer indicate the active/inactive status of a single pathway in a biological system, whereas the hidden layers show the interactive effects of multiple pathways. The deeper hidden layer expresses the higher-level representations of biological pathways. The connections in the hidden layers are sparsely established by sparse coding, so that model interpretation can be possible.

#### Clinical layer

The clinical layer introduces clinical data to the model separately from genomic data to capture clinical effects. The independent pipeline for clinical data also prevents the genomic data, of relatively higher-dimension, from dominating the effect of the model. In Cox-PASNet, the complex genomic effects of gene expression data are captured from the gene layer to the hidden layers, whereas the clinical data are directly introduced into the output layer, along with the highest-level representation of genomic data (i.e., node values on the last hidden layer). Therefore, Cox-PASNet takes the effects of genomic data and clinical data into account separately in the neural network model. If richer clinical information is available, multiple hidden layers in the clinical layers can be considered.

#### Cox layer

The Cox layer is the output layer that has only one node. The node value produces a linear predictor, a.k.a. Prognostic Index (PI), from both the genomic and clinical data, which is introduced to a Cox-PH model. Note that the Cox layer has no bias node according to the design of the Cox model.

Furthermore, we introduce sparse coding, so that the model can be biologically interpretable and mitigate the overfitting problem. In a biological system, a few biological components are involved in biological processes. The sparse coding enables the model to include only significant components, for better biological model interpretation. Sparse coding is applied to the connections from the gene layer to the last hidden layer by mask matrices. The sparse coding also makes the model much simpler, having many fewer parameters, which relieves overfitting problem.

### Objective function

Cox-PASNet optimizes the parameters of the model, ***Θ***={***β***,**W**}, by minimizing the average negative log partial likelihood with *L*^2^ regularization, where ***β*** is the Cox proportional hazards coefficients (weights between the last hidden layer and the Cox layer) and **W** is a union of the weight matrices on the layers before the Cox layer. The objective function of the average negative log partial likelihood is defined as follows:

1 $$\begin{array}{*{20}l} \ell(\boldsymbol{\Theta}) = &-\frac{1}{n_{E}}\sum_{i \in E}\left(\mathbf{h}_{i}^{I}\boldsymbol\beta - \text{log}\!\!\sum_{j \in R(T_{i})}\exp(\mathbf{h}_{j}^{I}\boldsymbol\beta)\right) \,+\, \lambda(\| \boldsymbol{\Theta}\|_{2}),  \end{array} $$

where **h**^*I*^ is the layer that combines the second hidden layer’s outputs and the clinical inputs from the clinical layer; *E* is a set of uncensored samples; and *n*_*E*_ is the total number of uncensored samples. *R*(*T*_*i*_)={*i*|*T*_*i*_≥*t*} is a set of samples at risk of failure at time *t*; ∥***Θ***∥_2_ is the *L*^2^-norms of {**W**,***β***} together; and *λ* is a regularization hyper-parameter to control sensitivity (*λ*>0).

**Fig. 7:**
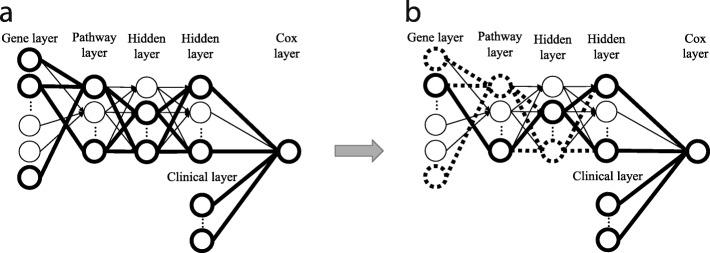
Training of Cox-PASNet with high-dimensional, low-sample size data. **a** A small sub-network is randomly chosen by a dropout technique in the hidden layers and trained. **b** Sparse coding optimizes the connections in the small network

We optimize the model by partially training small sub-networks with sparse coding. Training a small sub-network guarantees feasible optimization, with a small set of parameters in each epoch. The overall training flow of Cox-PASNet is illustrated in Fig. 7.

Initially, we assume that layers are fully connected, except between the gene layer and the pathway layer. The initial parameters of weights and biases are randomly initialized. For the connections between the gene layer and pathway layer, sparse connections are forced by the bi-adjacency matrix, which is a mask matrix that indicates the gene memberships of pathways. A small sub-network is randomly chosen by a dropout technique in the hidden layers, excluding the Cox layer (Fig. 7a). Then the weights and the biases of the sub-network are optimized by backpropagation. Once the training of the sub-network is complete, sparse coding is applied to the sub-network by trimming the connections within the small network that do not contribute to minimizing the loss. Figure 7b illustrates the sparse connections, and the nodes dropped by sparse coding are marked with bold and dashed lines. The algorithm of Cox-PASNet is briefly described in Algorithm 1.


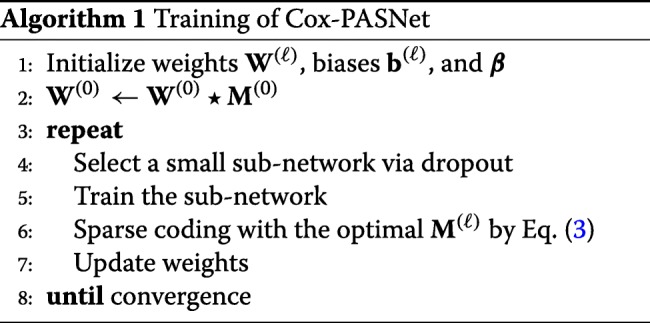


### Sparse coding

Sparse coding is proposed to make the connections between layers sparse for the model interpretation. Sparse coding is implemented by a mask matrix on each layer in the model. A binary mask matrix **M** determines the sparse connections of the network, where an element indicates whether the corresponding weight is zero or not. Then, the outputs, **h**^(*ℓ*)^, in the *ℓ*-th layer are computed by:

2 $$ \mathbf{h}^{(\ell +1)} = a\left((\mathbf{W}^{(\ell)}\star\mathbf{M}^{(\ell)})\mathbf{h}^{(\ell)}+\mathbf{b}^{(\ell)}\right),   $$

where ⋆ denotes an element-wise multiplication operator; *a*(·) is a nonlinear activation function (e.g., sigmoid or Tanh); and **W**^(*ℓ*)^ and **b**^(*ℓ*)^ are a weight matrix and bias vector, respectively (1≤*ℓ*≤*L*−2, and *L* is the number of layers).

In particular, an element of the binary mask matrix **M** is set to one if the absolute value of the corresponding weight is greater than threshold *s*^(*ℓ*)^; otherwise it is zero. The mask matrix between the gene layer and pathway layer (**M**^(0)^) is given from pathway databases, whereas other mask matrices (**M**^(*ℓ*)^,*ℓ*≠0) are determined by:

3 $$ \mathbf{M}^{(\ell)}=\mathbbm{1}(|\mathbf{W}^{(\ell)} | \geq s^{(\ell)}), \indent \ell \neq 0,   $$

where *s*^(*ℓ*)^ is the optimal sparsity level; and the function *𝟙*(*x*) returns one if *x* is true; otherwise it is zero. The optimal *s*^(*ℓ*)^ is heuristically estimated on each layer in the sub-network to minimize the cost function. In this study, we considered a finite set of sparsity levels in a range of **s**=[0,100), and computed scores. Note that a sparsity level of zero produces a fully-connected layer, whereas that of 100 makes disconnected layers. Then we approximated the cost function with respect to sparsity levels by applying a cubic-spline interpolation to the cost scores computed by the finite set of **s**. Finally, the sparsity level that minimizes the cost score was considered for the optimal sparsity level. The optimal *s*^(*ℓ*)^ is approximated on each layer, individually, in the sub-network. The individual optimization of the sparsity on each layer represents various levels of biological associations on genes and pathways.

## Data Availability

The datasets are publicly available and accessible at http://cancergenome.nih.gov. The open-source code of Cox-PASNet in PyTorch is available at https://github.com/DataX-JieHao/Cox-PASNet.

## Abbreviations

Adam: Adaptive moment estimation
Cox-EN: Cox elastic net
Cox-PASNet: Pathway-based sparse deep neural network for survival analysis
Cox-PH: Cox proportional hazards
GBM: Glioblastoma multiforme
H2: The second hidden layer
HDLSS: High-dimension, low-sample size
KPS: Karnofsky performance score
MSigDB: Molecular signatures database
OV: Ovarian serous cystadenocarcinoma
PI: Prognostic index
RSF: Random survival forest
SVM: Support vector machine
TCGA: The cancer genome atlas
